# Exploring the prognostic value of HK3 and its association with immune infiltration in glioblastoma multiforme

**DOI:** 10.3389/fgene.2022.1033572

**Published:** 2023-01-12

**Authors:** Yuling Yang, Xing Fu, Runsha Liu, Lijuan Yan, Yiping Yang

**Affiliations:** ^1^ Department of Radiation Oncology, Shaanxi Provincial Cancer Hospital, Xi’an Medical University, Xi’an, China; ^2^ Department of Radiation Oncology, Ankang Central Hospital, Ankang, China; ^3^ Clinical Research Center for Shaanxi Provincial Radiotherapy, Department of Radiation Oncology, Shaanxi Provincial Cancer Hospital, Xi’an, China

**Keywords:** GBM, prognosis, immune, HK3, macrophages

## Abstract

**Background:** Hexokinase 3 (HK3) is one of the key enzymes involved in glucose phosphorylation (the first step in most glucose metabolic pathways). Many studies have demonstrated the vital role of dysregulation of HK3 in several tumors. However, there is a need for in-depth characterization of the role of HK3 in glioblastoma multiforme (GBM).

**Methods:** All data were sourced from The Cancer Genome Atlas (TCGA) and Chinese Glioma Genome Atlas (CGGA). Kaplan-Meier analysis and univariate regression were applied for survival analysis. Gene set enrichment analysis (GSEA) was used for enrichment analysis. Tumor Immune Single Cell Hub (TISCH) database was applied for single-cell analysis. Tumor Immune Dysfunction and Exclusion (TIDE) analysis was applied to evaluate the immune response.

**Results:** HK3 expression was upregulated in GBM and correlated with poor prognosis. The high HK3 expression group was primarily enriched in adaptive immune response, chemokine signaling pathway, and cytokine-cytokine receptor interaction. The high HK3 expression group showed significantly greater enrichment of the majority of immune cells and immune-related pathways. HK3 showed significant correlation with most immune cells, especially macrophages (*p* < .001, R = .81). TISCH analysis showed that HK3 was predominantly expressed in macrophages in most cancers. HK3 showed significant correlation with most immune-related genes, such as PD-1 (*p* < .001, R = .41), PDL-1 (*p* < .001, R = .27), and CTLA-4 (*p* < .001, R = .29). TIDE analysis revealed that the low HK3 expression group has a lower TIDE score and may benefit from immunotherapy. Drug sensitivity analysis showed that patients with high HK3 expression frequently showed drug resistance.

**Conclusion:** HK3 was associated with poor prognosis and may serve as a biomarker of macrophages in GBM. HK3 was also associated with immune response and drug resistance. Our findings may provide novel insights for GBM immunotherapy.

## Introduction

Glioblastoma multiforme (GBM) is considered as the most frequent primary tumor of the nervous system (Ostrom et al., 2019). Patients with GBM have poor prognoses (median overall survival [OS]: 15 months; 5-year survival rate: <5%) (Tan et al., 2020). Several novel therapies, such as immune checkpoint inhibitors, anti-angiogenesis, tumor vaccines, and Tumor Treating Fields (TTFields), have been tried in GBM (Rodríguez-Camacho et al., 2022); however, most of these therapies are ineffective (Mun et al., 2018). Therefore, understanding the molecular mechanisms and exploring more effective targets for GBM are key research imperatives (Uddin et al., 2022).

Metabolic reprogramming is a core feature of tumors characterized by upregulation of glycolysis (Biswas, 2015; Pavlova and Thompson, 2016). Metabolic reprogramming plays an essential role in tumors (Pavlova and Thompson, 2016). Acyl-CoA-binding protein was found to drive GBM tumorigenesis by sustaining fatty acid oxidation (Duman et al., 2019). Isocitrate dehydrogenases (IDH)1/2 was found to drive GBM progression by producing an oncometabolite (Chang et al., 2019). Metabolic programming was shown to help maintain the proliferation of stem cell-like tumor cells in GBM (Suvà et al., 2014). Metabolic reprogramming has also been shown to be involved in the immune activity (Xia et al., 2021). Tumor depletion of glucose limits the metabolism of T cells, resulting in their diminished mTOR activity, glycolytic capacity, and interferon (IFN)-γ production (Chang et al., 2015).

Hexokinases are the key enzymes in metabolism (Cavalcante et al., 2016), including HK1, HK2, and HK3 (Wilson, 2003). High expression of HK1 has been shown to be associated with poor prognosis in the context of various tumors, such as colorectal and ovarian cancer (Graziano et al., 2017; Li T. et al., 2020; Jiang et al., 2021). A study demonstrated downregulation of HK1 in GBM (Wolf et al., 2011a). HK2 was shown to promote the motility and proliferation of human ovarian cancer cells by activating Akt1/p-Akt1 (Tian et al., 2022). Moreover, HK2 was shown to promote GBM progression by glycolysis (Wolf et al., 2011b). HK3 was found to be up-regulated in GBM; however, its role has not been extensively investigated.

In this study, we performed a comprehensive analysis of the role of HK3 in GBM using The Cancer Genome Atlas (TCGA), Chinese Glioma Genome Atlas (CGGA), Tumor Immune Single Cell Hub (TISCH), and Human Protein Atlas (HPA) databases. In particular, we performed *HK3* gene expression analysis, survival analysis, immune infiltration analysis, single-cell RNA sequencing analysis, and functional enrichment analysis to investigate the prognostic and immunological significance of HK3 in GBM.

## Materials and methods

### Datasets collection and pre-processing

RNA-sequencing data and associated clinical information of 33 types of cancers were sourced from the UCSC Xena (http://xena.ucsc.edu/). The CGGA-325 dataset was sourced from the CGGA (http://www.cgga.org.cn/) (Zhao et al., 2021). Samples with incomplete clinical information were excluded.

Genotype-tissue expression (GTEx) was available from the UCSC Xena. GTEx includes RNA-sequencing data of 207 cerebral cortex samples. After merging the TCGA-GBM and GTEx, the “normalizeBetweenArrays” function of the “limma” package was applied to eliminate the batch effect (Wu et al., 2022). All RNA-sequencing data were normalized (Fragments Per Kilobase Million, FPKM) and log2^(FPKM+1)^ transformed.

### Survival analysis

Univariate cox regression analysis was used to determine the prognostic value of HK3. Kaplan-Meier survival curves were plotted using the “surv” and “survminer” R packages, and between-group differences in survival were assessed using the log-rank test.

### Immune-related analysis

The enrichment scores of 16 immune cells and 13 immune-related pathways were computed using the “GSVA” and “GSEABase” R packages. The correlation between gene expression and immune cells was assessed using the TIMER 2.0 web application (http://timer.cistrome.org/) (Li Y. et al., 2020). The correlation between HK3 and immune-related genes was determined using Spearman’s correlation analysis (Zhu et al., 2021). Tumor Immune Dysfunction and Exclusion (TIDE) analysis includes MSI Expr Sig, Dysfunction, Exclusion, and TIDE scores, which were computed by uploading gene expression data through the web application (http://tideere.dfci.harvard.edu). Patients with low TIDE scores may benefit from immunotherapy (Fu et al., 2020).

### HK3 expression in immune cells

The “RNA immune cell” is a part of the Human Protein Atlas (HPA) which was applied to explore the gene expression in immune cells (https://www.proteinatlas.org/) (Karlsson et al., 2021). In this part, the Monaco dataset includes 29 immune cells, and the Schmiedel dataset includes 15 immune cells in peripheral blood (Schmiedel et al., 2018; Monaco et al., 2019). The TISCH is a convenient web application for single-cell analysis (http://tisch.comp-genomics.org/home/), which includes 190 single-cell datasets (Sun et al., 2021).

### Drug sensitivity analysis

The “OncoPredict” R package based on Genomics of Drug Sensitivity in Cancer (www.cancerrxgene.org/) was used to calculate the half-maximal inhibitory concentration (IC50) of drugs (Maeser et al., 2021).

### Cell lines and culture

The human glioblastoma cell lines (U87, A172, and U373) and normal human astrocytes (NHA) cell line SVGp12 employed in the study were purchased from American Type Culture Collection. Cells were cultured in Dulbecco’s modified Eagle’s medium (DMEM; HyClone, Logan, United States) with 10% fetal bovine serum (FBS; Gibco, NY, United States) and 1% penicillin-streptomycin (HyClone, Logan, United States) in 37°C incubators with 5% CO_2_.

### RNA isolation and RT-PCR

Total RNA was extracted using the RNAfast200 kit (Fastagen, China) according to the manufacturer’s instructions, and RNA concentration was quantified using NanoDrop 3,000 (ThermoFisher, United States). Then, 1.0 μg of total RNA in a 20 μL reaction system was reverse transcribed into cDNAs using Evo M-MLV RT Kit with gDNA Clean for qPCR (Accurate Biotechnology, China). qRT–PCR was carried out using 2 × RealStar Green Fast Mixture (GeneStar Technology, China). β-tubulin expression was used as the internal reference. The 2^−ΔΔCT^ method was applied to calculate the relative expression of *HK3*. The primer sequences were as follows:

HK3 5′-AGGGTATGGTCGAAGGTGGTCAG-3′(forward) and 5′-GTGGCAGTGCTGGACGAAGAC-3′(reverse); β-tubulin 5′-ACCTGATGTATGCCAAGCGT-3′(forward) and 5′-AGCTGAAATTCTGGGAGCATGA-3′(reverse).

### Statistical analysis

All statistical analyses were performed using R software (version 4.2.1). Spearman’s test was used for correlation analysis. *p* values <.05 were considered indicative of statistical significance.

## Results

### Expression of HK3 in cancers

**FIGURE 1:**
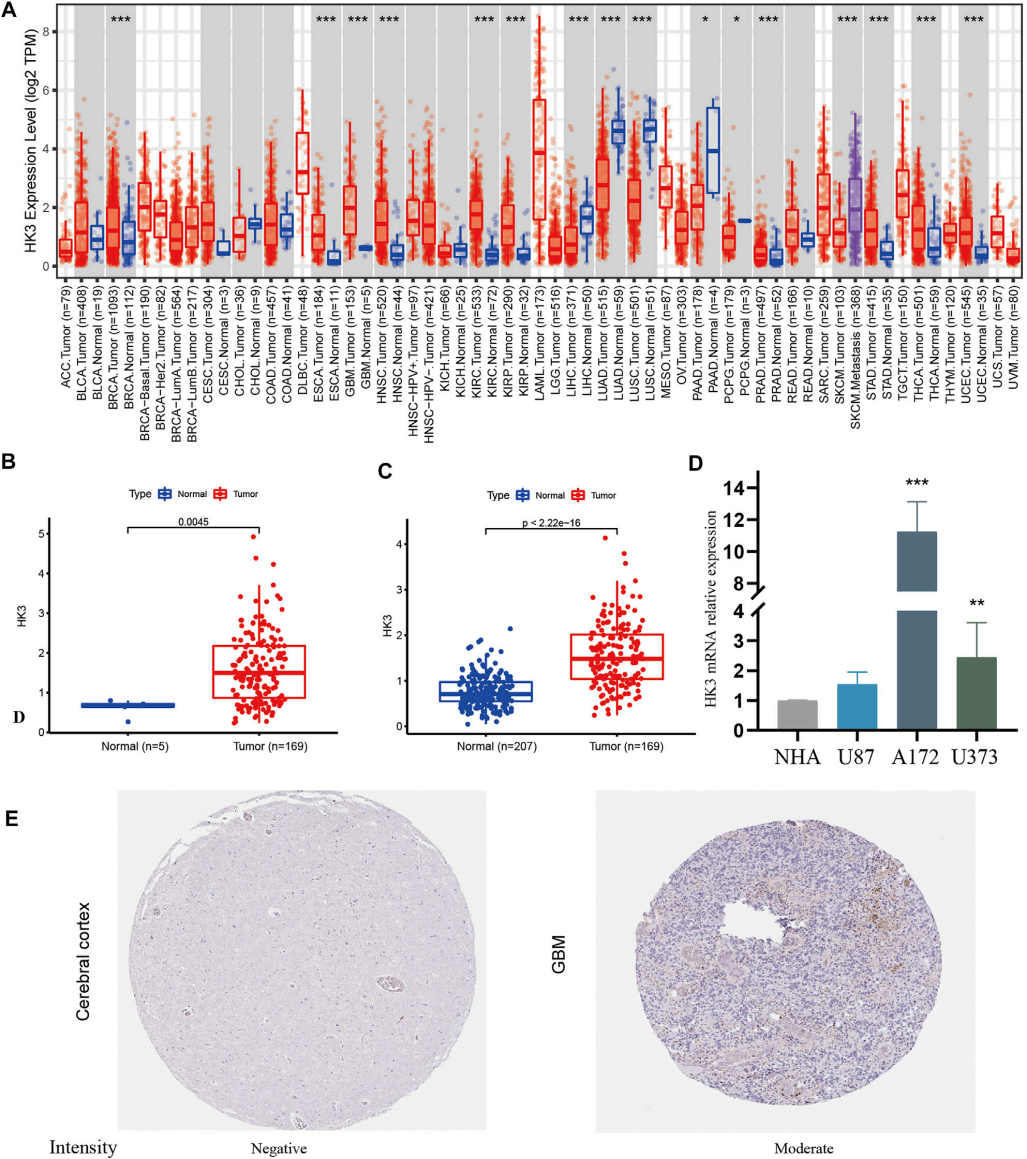
HK3 expression in cancers. **(A)** HK3 expression between tumor and normal samples at the pan-cancer level by the TIMER2.0; **(B)** HK3 expression between GBM and normal samples in the TCGA-GBM; **(C)** HK3 expression between GBM samples (TCGA-GBM) and normal samples (GTEx). **(D)** HK3 mRNA expression between NHA cells and U87, A172, and U373 cells; **(E)** Protein encoded by HK3 between GBM and normal tissue in the HPA (*p < .05; **p < .01; ***p < .001).

HK3 expression was explored at the pan-cancer level using TIMER2.0. The results showed dysregulation of HK3 expression in 14 types of human cancers (Figure 1A). HK3 mRNA expression in GBM samples was significantly higher than that in normal samples (Figures 1B, C). qRT-PCR showed that HK3 mRNA expression was significantly higher in A172 and U373 cells than in NHA cells, but not elevated in U87 cells (Figure 1D). The protein encoded by HK3 was also upregulated in GBM (Figure 1E).

### Prognostic value of HK3 in cancers

**FIGURE 2:**
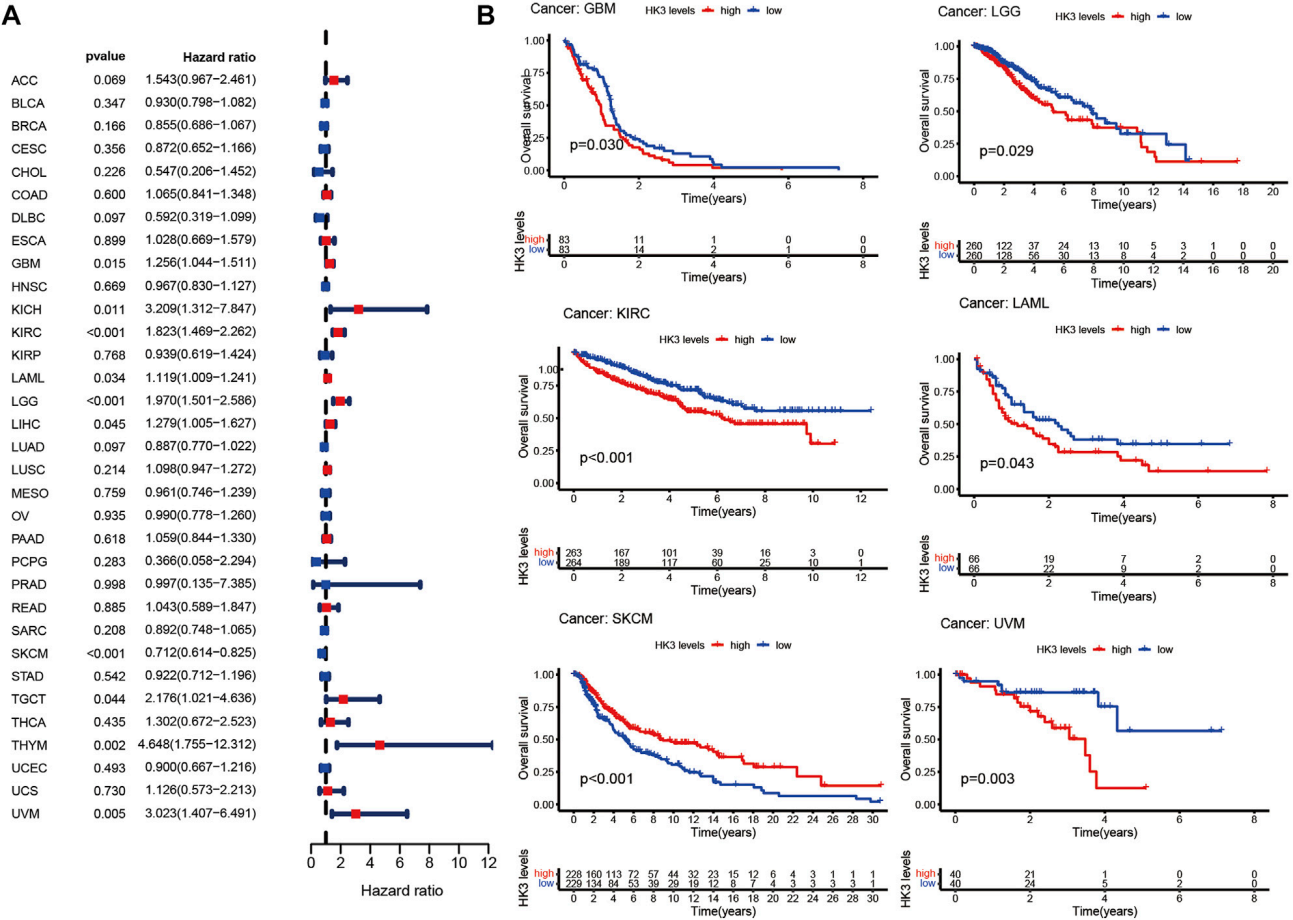
HK3 expression in 33 different types of tumors in the TCGA database and OS. **(A)** Forest plots of univariate Cox regression analysis; **(B)** Kaplan-Meier survival analysis.

**FIGURE 3:**
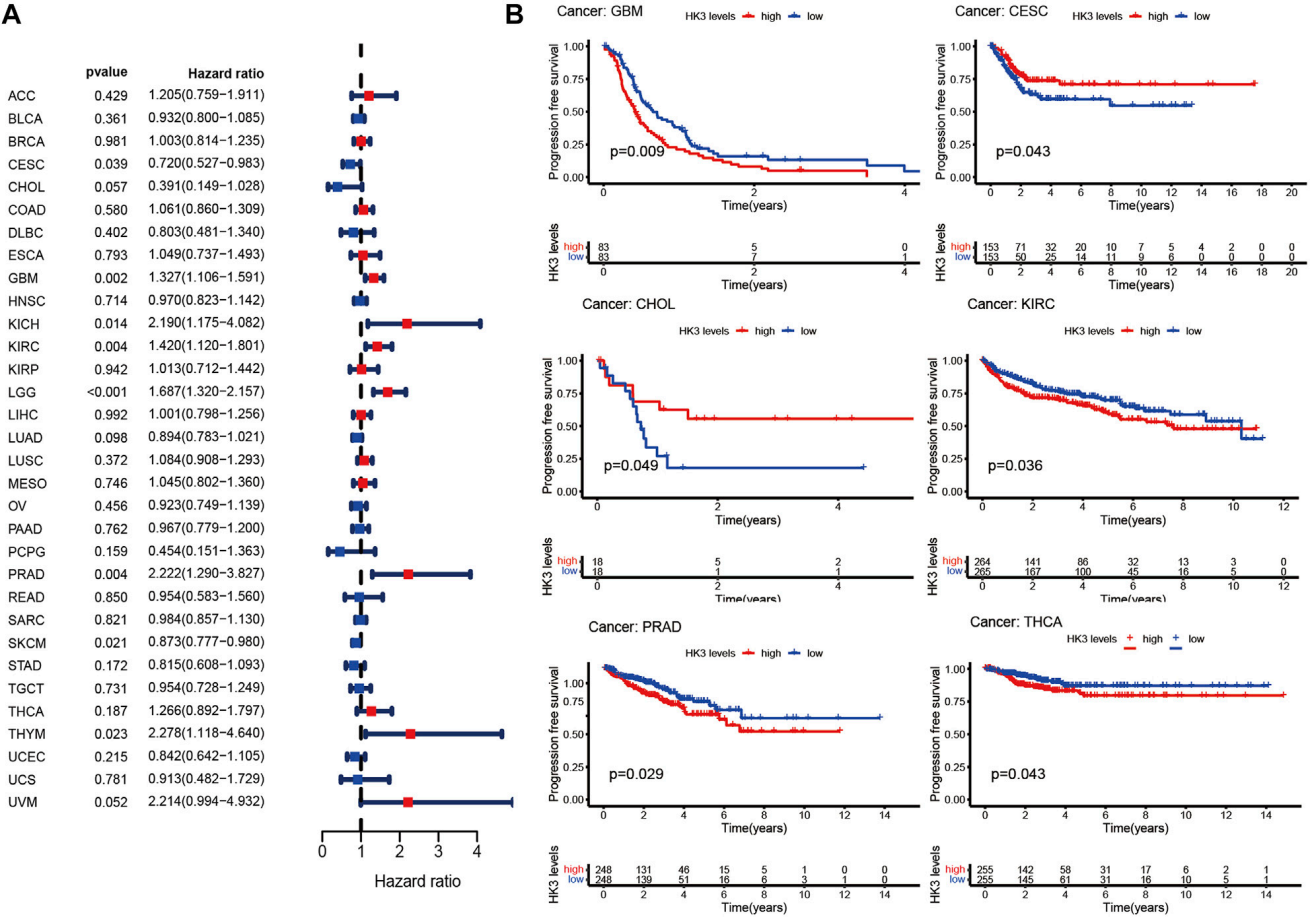
HK3 expression in 33 different types of cancers in the TCGA database and PFS. **(A)** Forest plots of univariate Cox regression analysis; **(B)** Kaplan-Meier survival analysis.

The prognostic value of HK3 is tumor-specific. Univariate Cox regression analysis showed that the expression of HK3 was associated with poor OS in GBM, kidney renal clear cell carcinoma (KIRC), brain lower grade glioma (LGG), thymoma (THYM), uveal melanoma (UVM), kidney chromophobe (KICH), acute myeloid leukemia (LAML), testicular germ cell tumors (TGCT), and liver hepatocellular carcinoma (LIHC), but associated with favorable OS in skin cutaneous melanoma (SKCM) (Figure 2A). Kaplan-Meier analysis showed that high expression of HK3 was associated with poor OS in GBM, LGG, KIRC, LAML, and UVM, but associated with favorable OS in SKCM (Figure 2B). The HK3 expression was also associated with poor OS of GBM in the CGGA-325 dataset (*p* < .05, Supplementary Figure S1). The results for progression-free survival (PFS) are illustrated in Figures 3A, B. These results suggested the prognostic value of HK3 in different tumors, and high expression of HK3 was usually associated with poor survival.

### Function enrichment analysis in GBM

**FIGURE 4:**
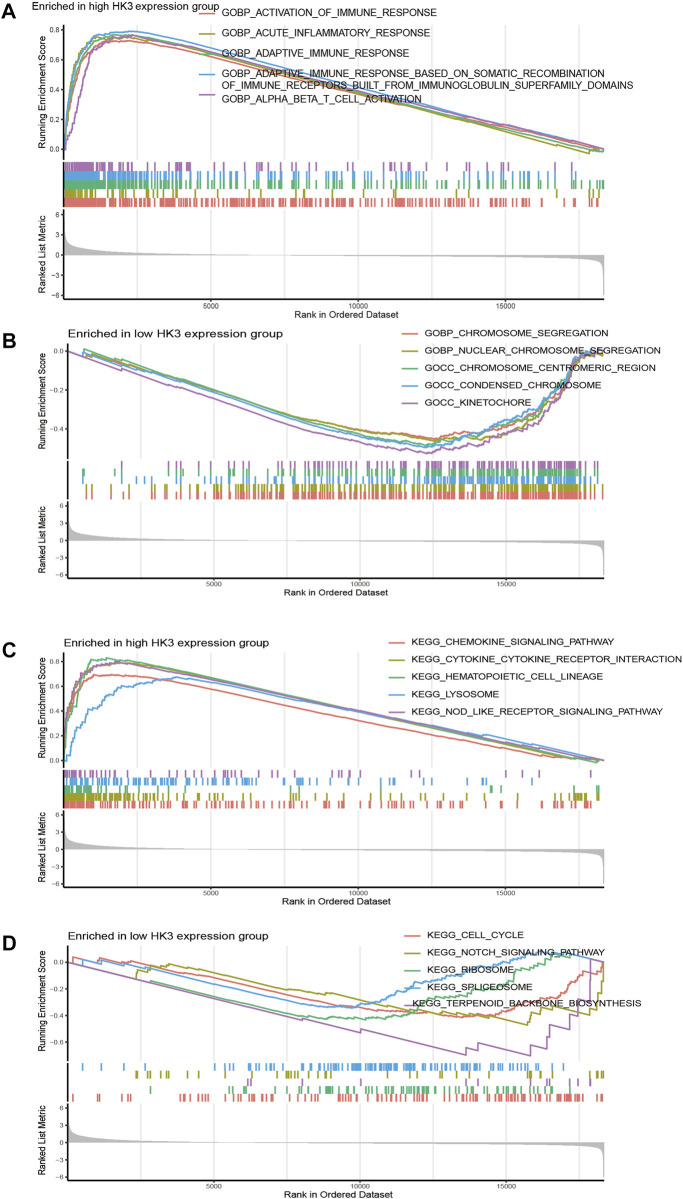
Function enrichment analysis in GBM. **(A,B)** The top five GO items enriched in the high- and low HK3 expression groups. **(C,D)** The top five KEGG items enriched in the high- and low HK3 expression groups.

To elucidate the biological function and pathways with HK3 involvement, we performed enrichment analysis in the TCGA-GBM. The top five gene ontology (GO) items enriched in the high HK3 expression group were activation of the immune response, acute inflammatory response, adaptive immune response, adaptive immune response based on somatic recombination of immune receptors built from immunoglobulin superfamily domains, and alpha-beta T cell activation (Figure 4A). In comparison, the top five GO items enriched in the low expression HK3 group were chromosome segregation, nuclear chromosome segregation, condensed chromosome, chromosome centromeric region, and kinetochore (Figure 4B). The top five KEGG items enriched in the high HK3 expression group were the chemokine signaling pathway, cytokine-cytokine receptor interaction, hematopoietic cell lineage, lysosome, and nod like receptor signaling pathway (Figure 4C). The top five KEGG items enriched in the low HK3 expression group were cell cycle, ribosome terpenoid backbone, biosynthesis, notch signaling pathway, and spliceosome (Figure 4D).

### Relationship between HK3 and immune characteristics in GBM

**FIGURE 5:**
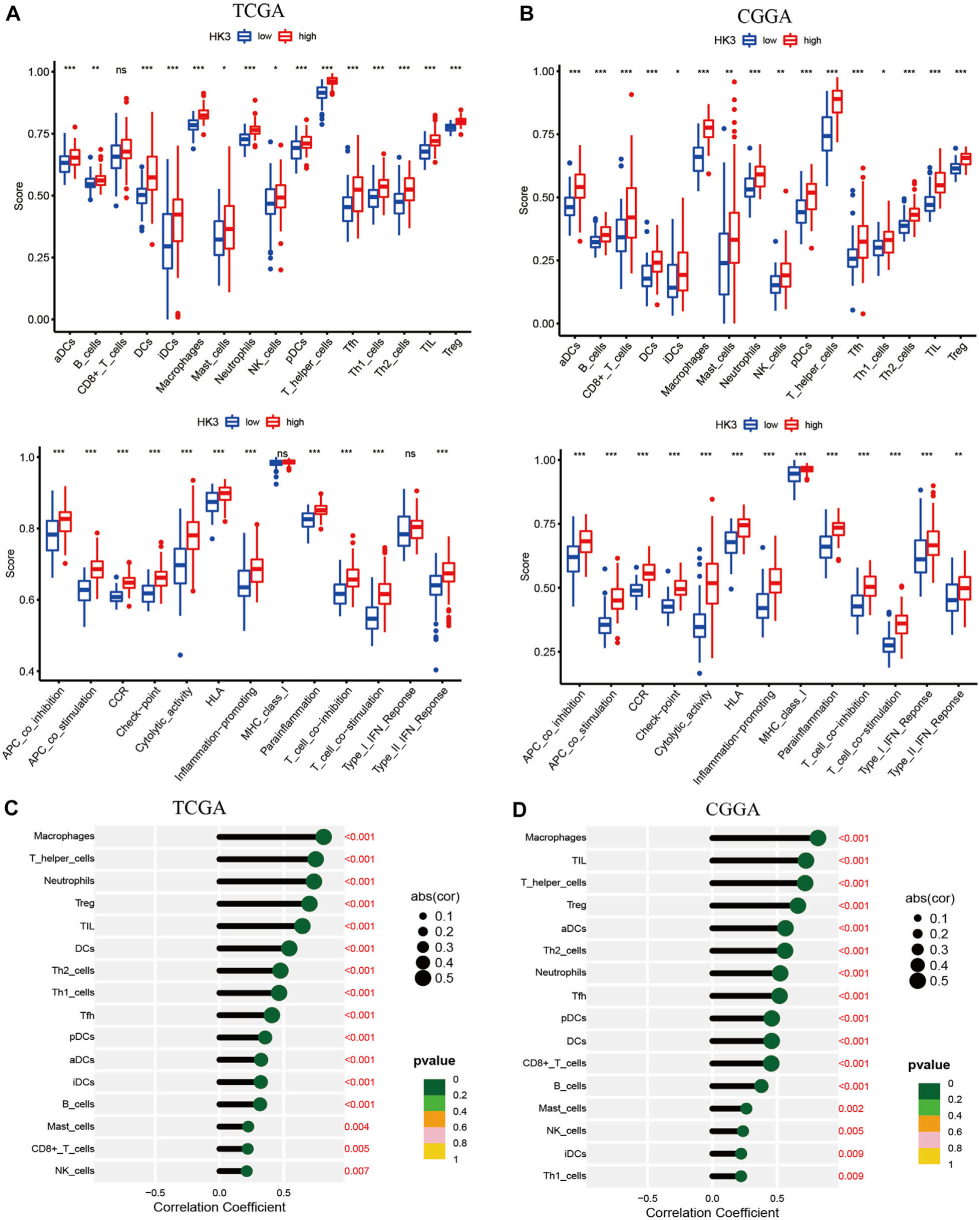
Relationship between HK3 and immune activity in GBM. Enrichment scores of 16 immune cells and 13 immune-related pathways between high- and low HK3 expression groups in the TCGA **(A)** and CGGA **(B)** cohorts. Correlation between HK3 expression and immune cell infiltration in the TCGA **(C)** and CGGA **(D)** cohorts. (*p < .05; **p < .01; ***p < .001).

We next performed GSEA analysis to investigate the immunological significance of HK3. In the TCGA and CGGA cohorts, the high HK3 expression group showed significantly greater enrichment of the majority of immune cells and immune-related pathways compared to the low HK3 expression group (Figures 5A, B). HK3 expression showed a significant correlation with immune cell infiltration in both cohorts (Figures 5C, D) (Supplementary Table S1). Interestingly, among all genes of GBM, HK3 showed the strongest correlation with macrophages (*p* < .001, R = .81) (Supplementary Table S2). Furthermore, increased macrophage infiltration was associated with poor OS in GBM in the TCGA and CGGA cohorts (Supplementary Figure S2). Analysis of TIMER 2.0 database showed that HK3 expression was associated with M2 macrophages, but not M1 (Supplementary Figure S3). HK3 showed a significant association with macrophage M2 polarization-related genes, such as CD163, VSIG4, MS4A4A, ITGAM, MRC1, and ITGAX (Supplementary Figure S3).

### Association of HK3 with macrophages

**FIGURE 6:**
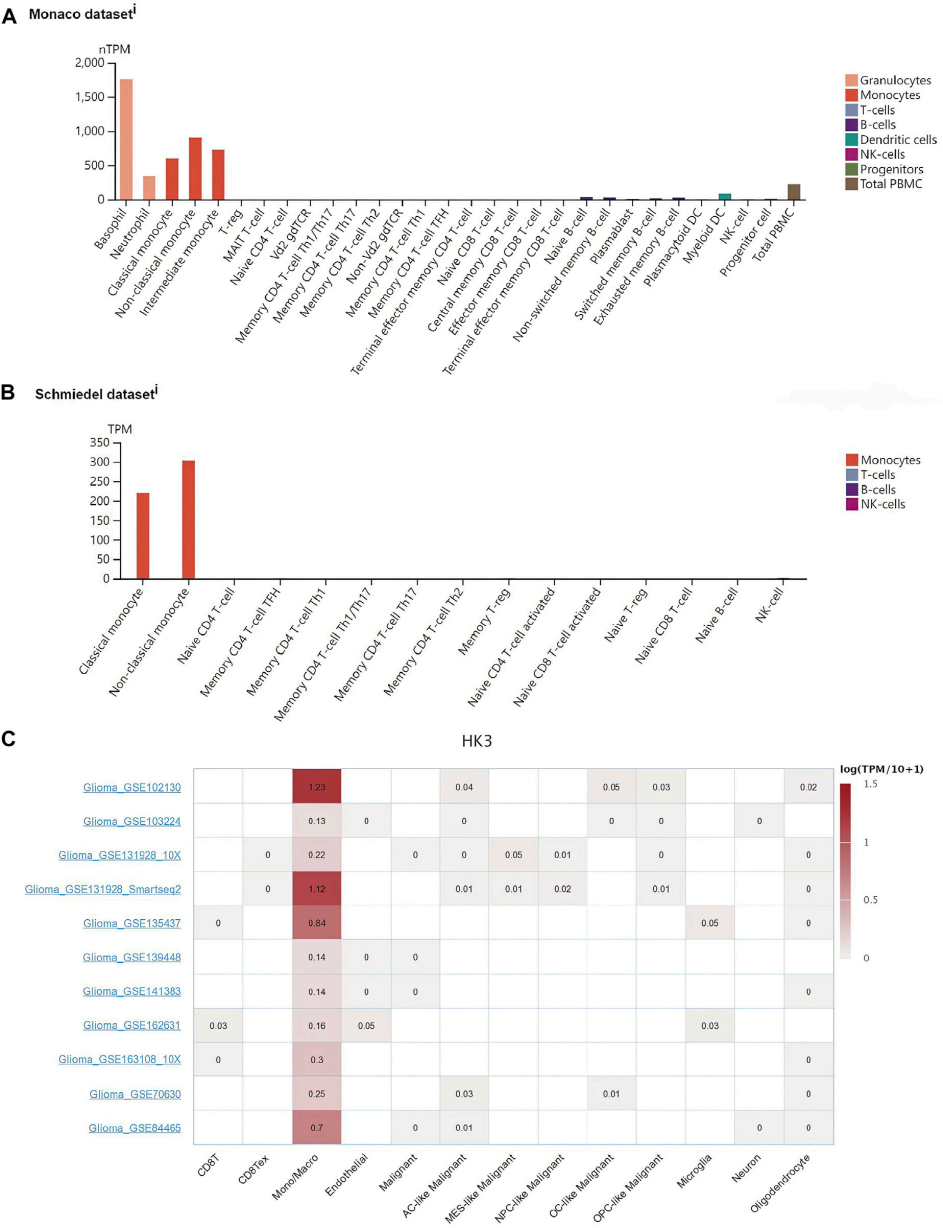
Association of HK3 expression with macrophages. HK3 expression in macrophages in the Monaco dataset **(A)** and Schmiedel dataset **(B)**. **(C)** HK3 expression in immune cells in GBM *via* the TISCH database.

Since the expression of HK3 showed a significant association with macrophages (*p* < .001, R = .81), we further explored whether HK3 expression in macrophages was specific. We investigated the HK3 expression in immune cells in plasma through the HPA. In the Monaco dataset, HK3 was predominantly expressed in basophils, neutrophils, and monocytes/macrophages (Figure 6A). In the Schmiedel dataset, HK3 was predominantly expressed in monocytes/macrophages (Figure 6B). We further explored the expression of HK3 at the single cell level through the TISCH. HK3 was found to be predominantly expressed in macrophages in GBM (Figure 6C). HK3 was also predominantly expressed in macrophages in ovarian serous cystadenocarcinoma (OV), pancreatic adenocarcinoma (PAAD), SKCM, LIHC, non-small cell lung cancer (NSCLC), head and neck squamous cell carcinoma (HNSC), KIRC, carcinoma of colon and rectum (CRC), breast invasive carcinoma (BRCA), chronic lymphocytic leukemia (CLL), and cervical squamous cell carcinoma and endocervical adenocarcinoma (CESC) (Supplementary Figures S4–S7).

### Co-expression of HK3 with immune-related genes

**FIGURE 7:**
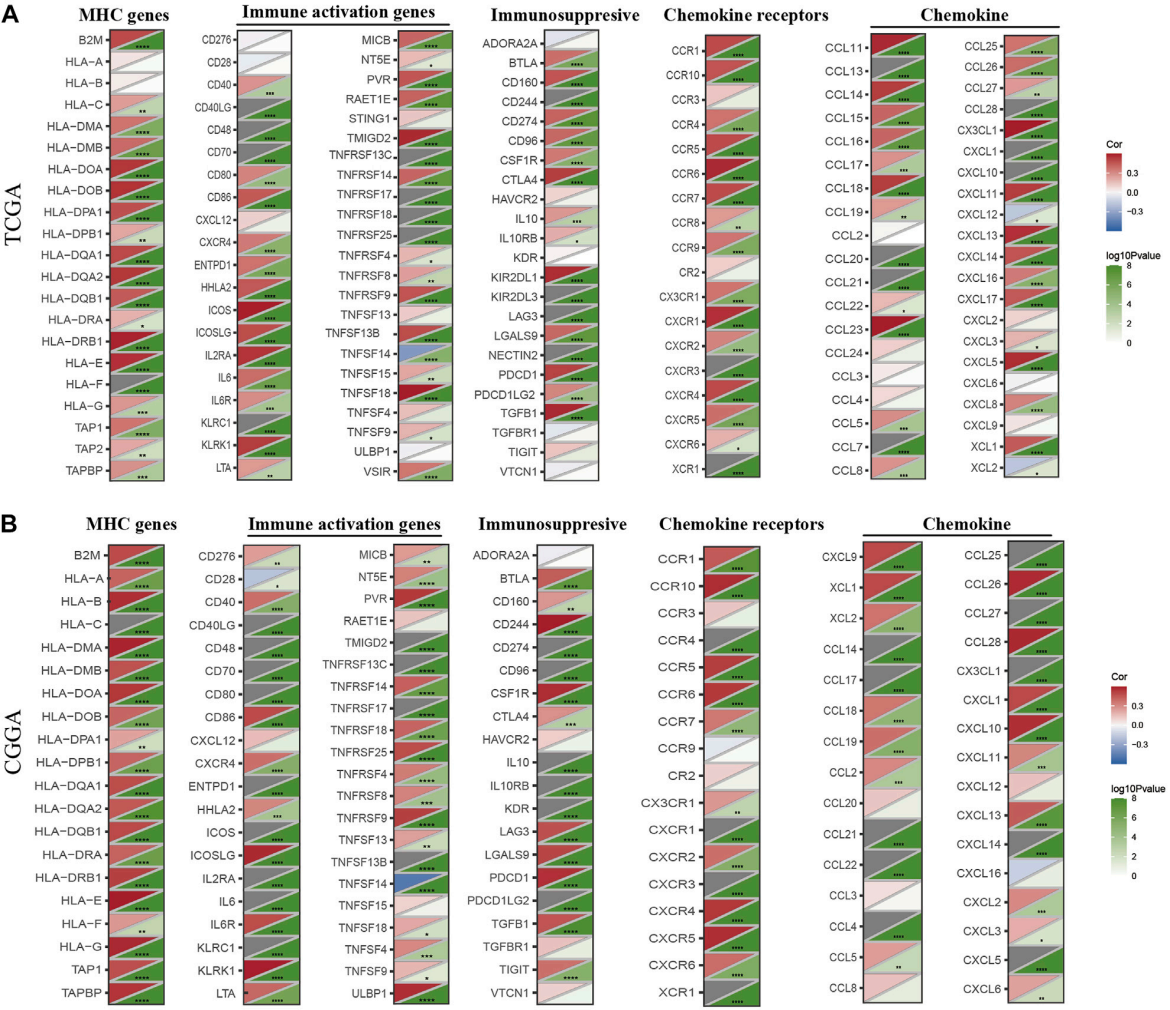
Co-expression of HK3 with immune-associated genes. Co-expression between HK3 and MHC genes, immune-activated genes, immunosuppressive genes, chemokines, and chemokines receptors in the TCGA **(A)** and CGGA **(B)** cohorts. (*p < .05, **p < .01, ***p < .001).

We next assessed the co-expression of HK3 with immune-related genes. The results showed a positive correlation of HK3 with most major histocompatibility complex (MHC), immunosuppressive, immune activation, chemokine, and chemokine receptor genes in the TCGA and CGGA cohorts (Figures 7A, B), such as PD-1 (*p* < .001, R = .41), PDL-1 (*p* < .001, R = .27), and CTLA-4 (*p* < .001, R = .29) (Supplementary Table S3).

### Association of HK3 with immune response and drug sensitivity

**FIGURE 8:**
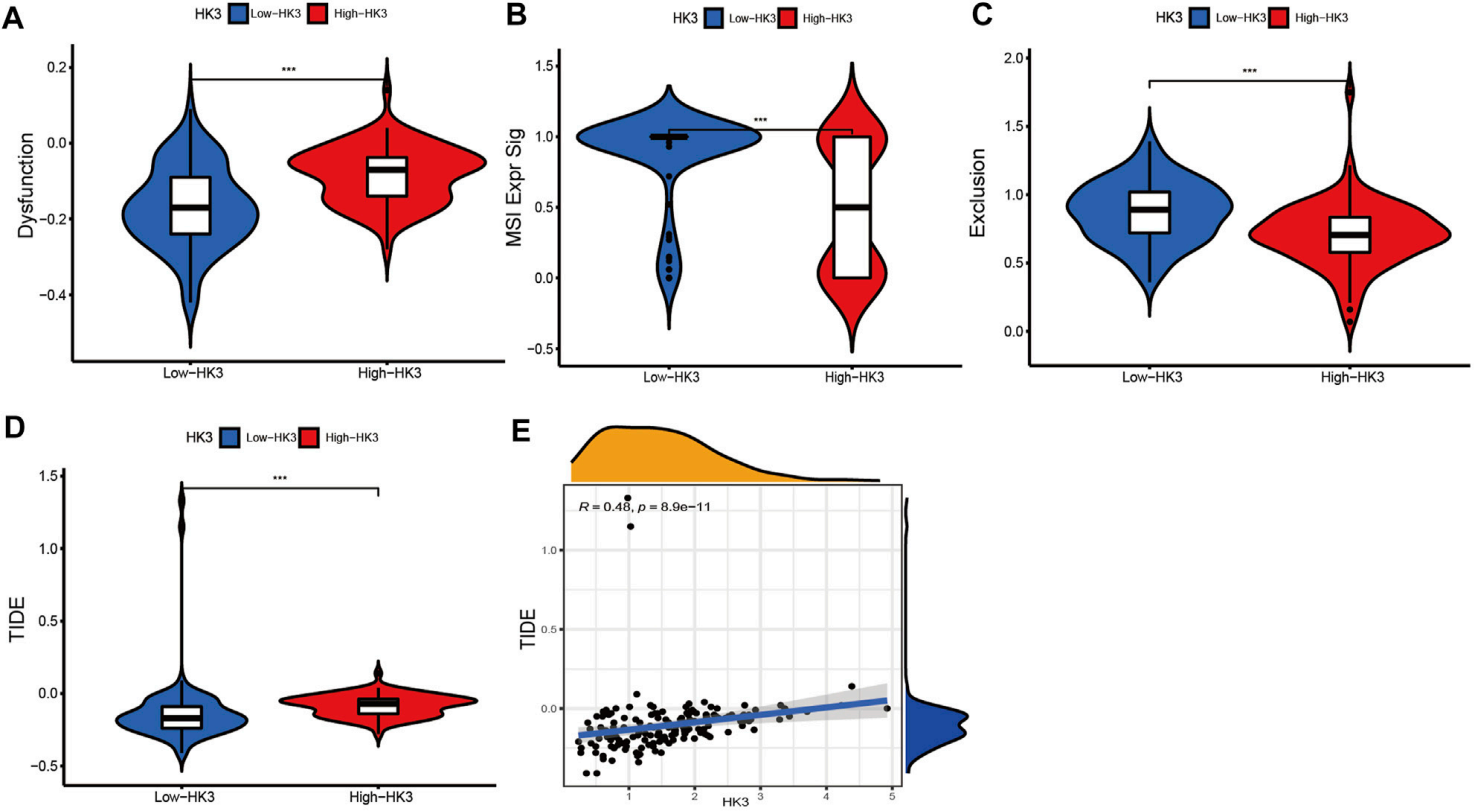
Relationship between HK3 and immune response. **(A–D)** Dysfunction-score, MSI Expr Sig-score, Exclusion-score, and TIDE-score between high- and low HK3 expression group; **(E)** Correlation of HK3 with TIDE-score (*p < .05; **p < .01; ***p < .001).

**FIGURE 9:**
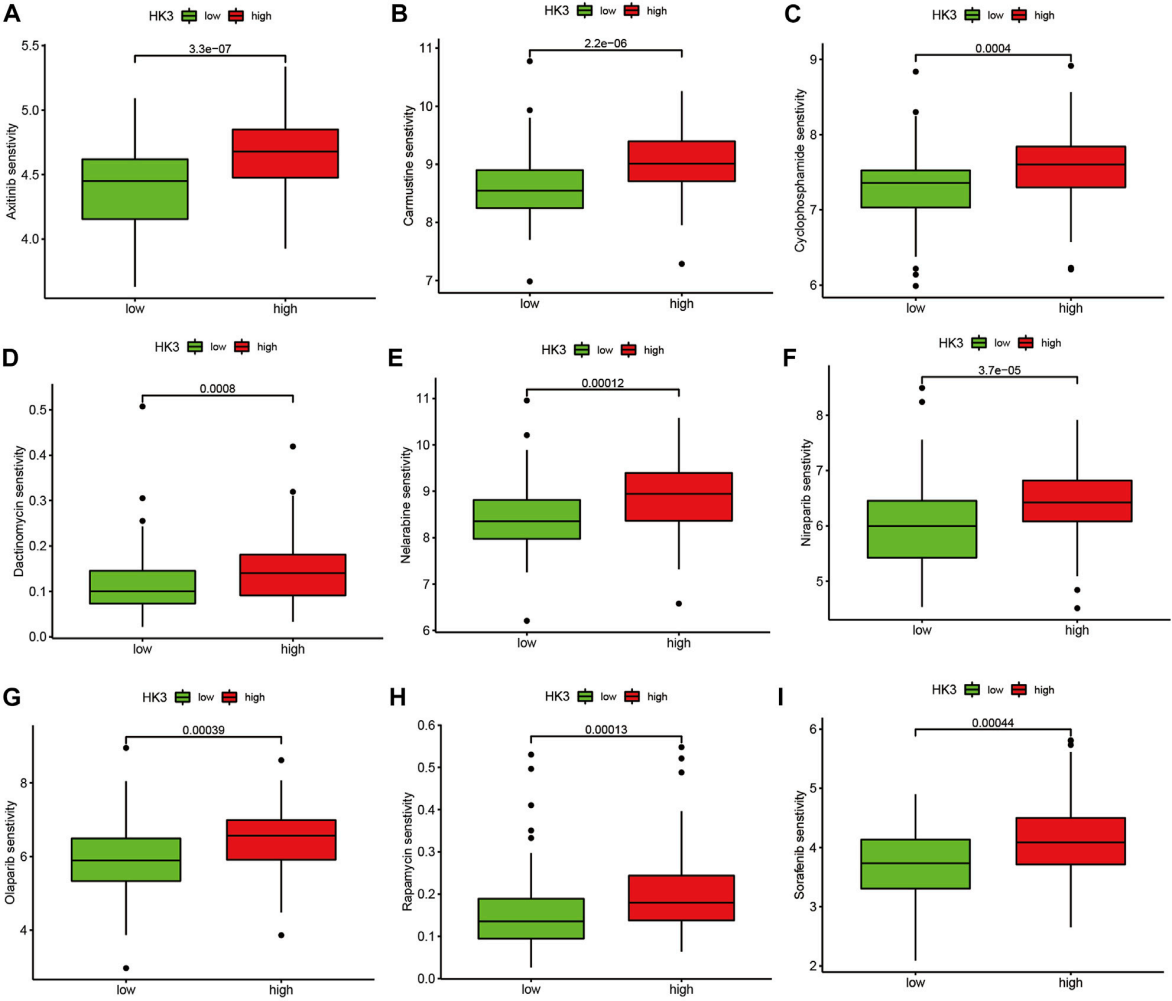
Drug sensitivity analysis. Comparison of the IC50 value of drugs between the high and low HK3 expression groups in the TCGA cohort. **(A)** Axitinib, **(B)** Carmustine, **(C)** Cyclophosphamide, **(D)** Dactinomycin, **(E)** Nelarabine, **(F)** Niraparib, **(G)** Olaparib, **(H)** Rapamycin, **(I)** Sorafenib.

We applied the TIDE analysis to explore the association between HK3 expression and the immune response. In the TCGA cohort, the Exclusion-score and the MSI Expr Sig-score in the low HK3 expression group were higher than that in high HK3 expression group, while Dysfunction-score and TIDE-score were lower in the low HK3 expression group (Figures 8A–D). HK3 showed a significant association with the TIDE score (*p* < .001, R = .48) (Figure 8E), and its correlation value ranked in the top 5% of all genes in GBM (Supplementary Table S4). Drug sensitivity analysis showed that the high HK3 expression group was frequently associated with resistance to drugs, such as axitinib, carmustine, cyclophosphamide, dactinomycin, and nelarabine (Figure 9).

## Discussion

GBM is a highly aggressive malignancy and the current treatments do not lead to satisfactory outcomes. Therefore, identification of new therapeutic targets in the context of GBM is a key imperative (Koh et al., 2022). Metabolic reprogramming is crucial for cancer and has emerged as a promising therapeutic target (Martínez-Reyes and Chandel, 2021). HK3 is one of the key enzymes involved in metabolism, but few studies have focused on its role in GBM. In the present study, we performed a comprehensive analysis of the prognostic and immunological significance of HK3 in GBM. Our findings may provide a basis for future studies.

We first explored HK3 expression and its prognostic value at the pan-cancer level using the TCGA database. Our results demonstrated dysregulation of HK3 in several tumors and its frequent correlation with poor prognosis. In previous studies, HK3 was shown to promote colorectal cell proliferation through epithelial mesenchymal transition (Xu et al., 2021). HK3 was also shown to promote metastasis of colorectal cancer *via* the nuclear factor κB/Snail/Hexokinase-3 signaling axis (Wu et al., 2021). Moreover, HK3 was found to prevent apoptosis in colorectal cancer and melanoma cells (Kudryavtseva et al., 2016). Downregulation of HK3 expression impaired neutrophil differentiation and increased sensitivity to anthracyclines in acute promyelocytic leukemia (Federzoni et al., 2012). High expression of HK3 was associated with poor OS in kidney cancer (Zhang Y. et al., 2021).

We applied enrichment analysis to investigate the potential involvement of HK3 in the biological functions and pathways in GBM. Intriguingly, the top five GO and KEGG items enriched in the high HK3 expression group were all related to immune activity, such as immune response, adaptive immune response, chemokine signaling pathway (Ortiz Zacarías et al., 2021), and cytokine-cytokine receptor interaction (Zheng et al., 2022). Thus, we explored the relationship between HK3 and immune activity. We observed consistently greater enrichment of most immune cells and immune-related pathways in the high HK3 expression group. These results suggest the potential involvement of HK3 in shaping the GBM tumor microenvironment.

Tumor-associated macrophages (TAMs) account for half of all non-tumor cells in GBM (Charles et al., 2012). TAMs are heterogeneous cellular populations composed of microglia, blood-derived infiltrating macrophages, and monocytes that cross the compromised blood-brain barrier (Zhang H. et al., 2021). Infiltration of TAMs has been shown to be associated with poor OS in the context of most tumors, such as PDAC (Zhang X. et al., 2020), GBM (Huang et al., 2020), and BLCA (Wu et al., 2020). Studies have demonstrated a crucial role of TAMs in GBM. SLIT2/ROBO signaling in TAM drives GBM immunosuppression and vascular dysmorphia (Geraldo et al., 2021). Pleiotrophin secreted by TAMs was shown to promote PTPRZ1 signaling in GBM stem cells leading to tumor growth (Shi et al., 2017). Colony-stimulating factor 1 receptor (CSF-1R) was found to inhibit macrophage polarization and block glioma progression (Pyonteck et al., 2013). Macrophage-associated pgk1 phosphorylation promoted GBM aerobic glycolysis and tumorigenesis (Zhang et al., 2018). In the present study, we observed a significant correlation of HK3 with macrophages in the GBM (*p* < .001, R > .8); in addition, HK3 was predominantly expressed in macrophages in most types of cancers at the single-cell level. Thus, HK3 may serve as a biomarker for macrophages. In previous studies, HK3 expression was found to be 100-fold higher in macrophages and granulocytes compared to other immune cells (Seiler et al., 2022). HK3 was also identified as a biomarker of macrophages in clear cell renal cell carcinoma (Xu et al., 2021). Moreover, HK3 showed an association with M2 macrophages and T cell dysfunction in GBM (Ji et al., 2022).

Immune checkpoint inhibitors represent a great breakthrough in cancer treatment, but these drugs are not effective against GBM (Chokshi et al., 2021). Although anti-PD-1 treatment was shown to increase T cell activity in GBM, TAMs account for half of immune cells, and their immunosuppressive effects resulted in treatment failure (de Groot et al., 2020; Lee et al., 2021). Several preclinical and clinical studies have identified TAM as a promising target for cancer immunotherapy (Zhang S. Y. et al., 2020). Differentiation of CD34 progenitor cells into macrophages using macrophage colony stimulating factor (M-CSF) was shown to induce a 6-fold increase in HK3 expression (Seiler et al., 2022). The generation of central nervous system macrophages relies on the transcription factor PU.1 (Goldmann et al., 2016), and HK3 was considered as transcriptional target of PU.1 (Federzoni et al., 2012). Thus, HK3 may be involved in macrophage differentiation and could be a potential target for anti-TAMs; however, further studies are required to verify this hypothesis. In our study, patients with low HK3 expression had lower macrophage infiltration and TIDE scores, indicating that these patients may potentially benefit from immunotherapies.

Our study demonstrated increased expression of HK3 expression in GBM tissues and high expression of HK3 was associated with poor prognosis. HK3 showed a significant association with macrophage infiltration and may serve as a biomarker of macrophages. HK3 was also associated with immune response and drug-resistance. In conclusion, this study provides a comprehensive understanding of the role of HK3 in GBM, which may help provide novel insights for developing GBM immunotherapy. Our study has some limitations. The underlying mechanism of the relationship of HK3 with macrophage differentiation and immunotherapy is unclear. Further studies are required to explore this aspect.

## Conclusion

HK3 was associated with poor prognosis of GBM and may serve as a biomarker of macrophages in GBM. HK3 was also associated with immune response and drug resistance. Our findings provide novel insights for development of GBM immunotherapy.

## Data Availability

The original contributions presented in the study are included in the article/Supplementary Material, further inquiries can be directed to the corresponding author.

## Abbreviations

AML, Acute myeloid leukemia; AUCs, Area under the ROC curves; Brain LGG, lower grade glioma; BRCA, Breast invasive carcinoma; CRC, Carcinoma of colon and rectum; CESC; ES, Cervical squamous cell carcinoma and endocervical adenocarcinoma; CGGA, Chinese Glioma Genome Atlas; CLL, Chronic lymphocytic leukemia; GTEx, Genotype-Tissue Expression; GBM, Glioblastoma multiforme; HPA, Human Protein Atlas; ICIs, immune checkpoint inhibitors; KICH, Kidney chromophobe; HNSC, Head and Neck squamous cell carcinoma; KIRC, Kidney renal clear cell carcinoma, LIHC, Liver hepatocellular carcinoma; OV, Ovarian serous cystadenocarcinoma; OS, overall survival; PAAD, Pancreatic adenocarcinoma; ROC, Receiver operating characteristic; SKCM, Skin cutaneous melanoma; TGCT, Testicular germ cell tumors; TCGA, The Cancer Genome Atlas; THYM, thymoma; TIDE, Tumor Immune Dysfunction, and Exclusion; TISCH, Tumor Immune Single-cell Hub; UVM, Uveal melanoma.
