# Supplementary material for: Exploring the prognostic value of HK3 and its association with immune infiltration in glioblastoma multiforme
Source: Front Genet. 2023 Jan 12;13:1033572. doi: 10.3389/fgene.2022.1033572 (PMC9877303; doi:10.3389/fgene.2022.1033572)
Supplement: Supplementary file 10 [file Table10.DOCX]

**Supplementary Figures**

**Figure S1**

**
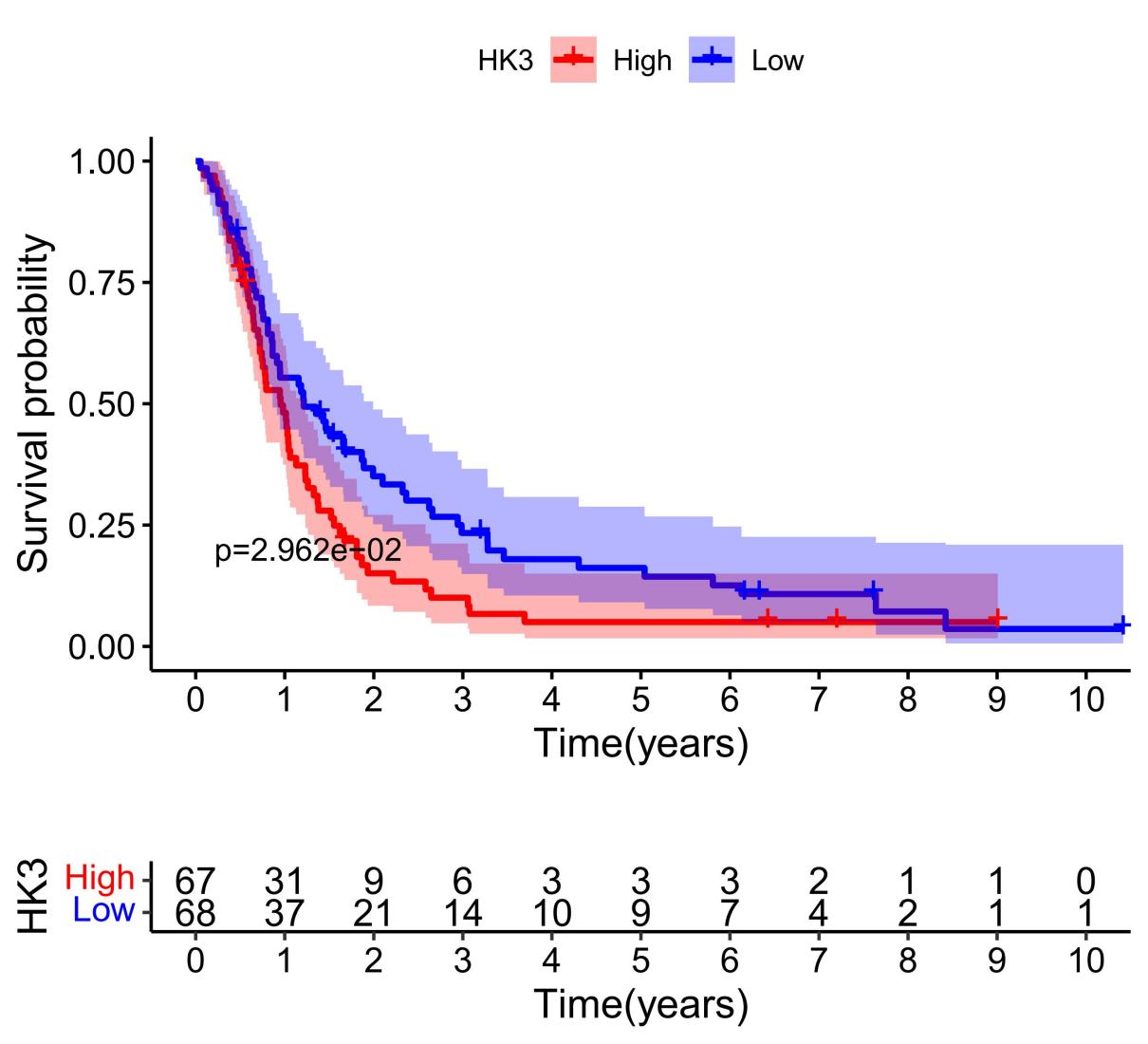
**

**Figure S1.** Kaplan-Meier survival analysis in the CGGA-325.

**Figure S2**

**
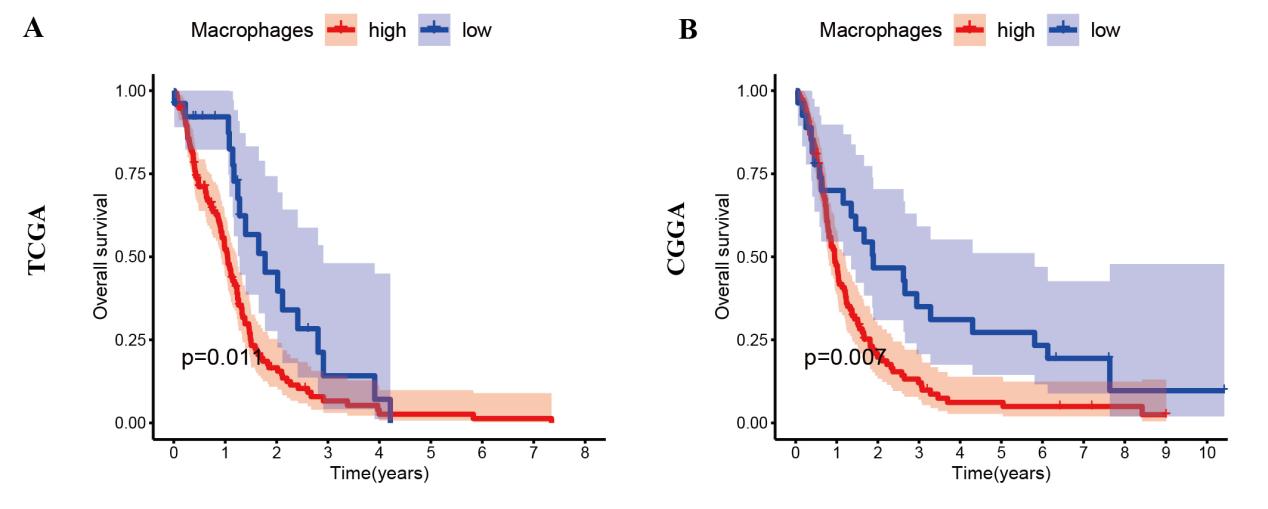
**

**Figure S2.** The "surv_cutpoint" function of the "survival" R package was applied to select the optimum value and classify patients into high- and low-macrophages groups. Kaplan-Meier survival analysis in the TCGA (A), and CGGA cohorts (B).

**Figure S3**

**
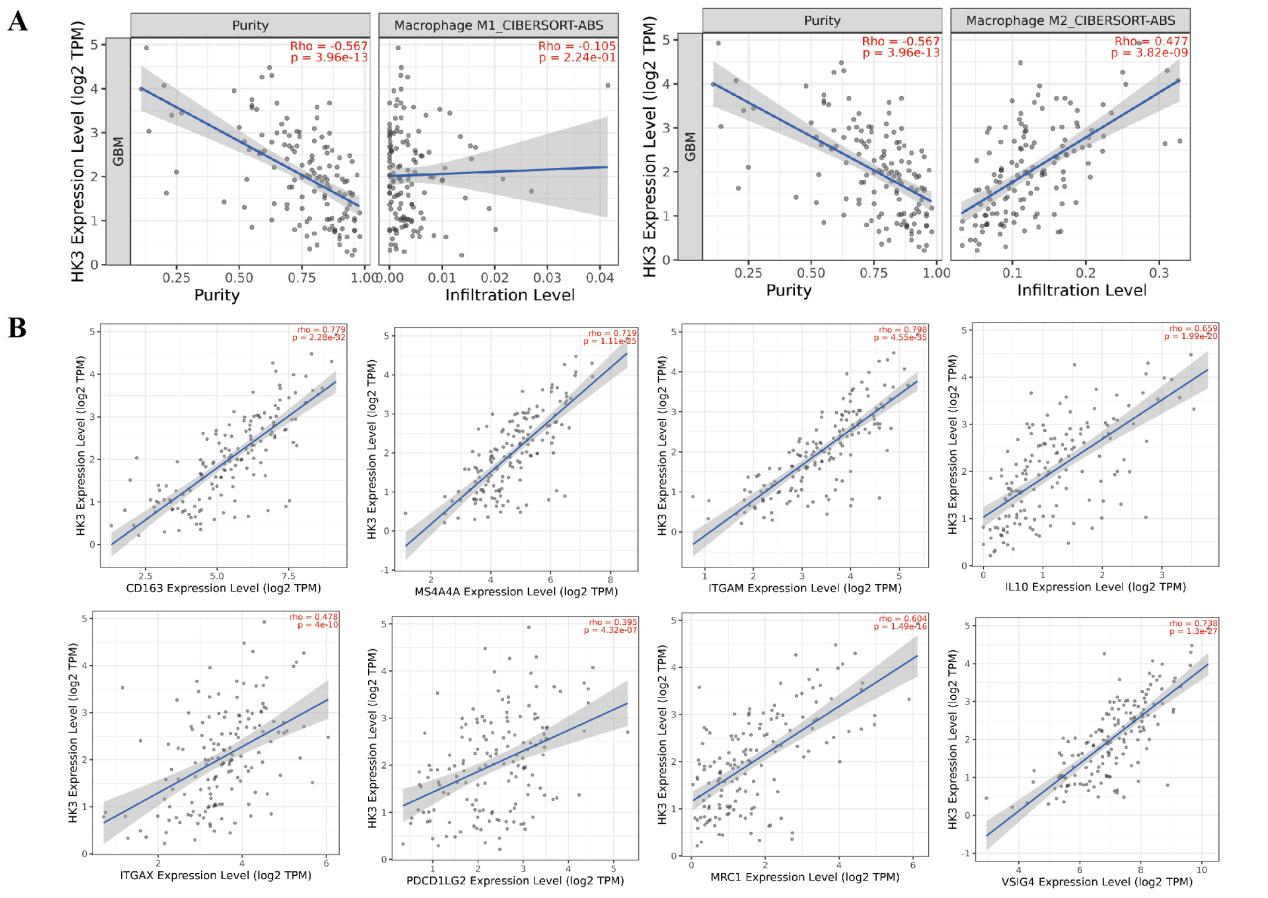
**

**Figure S3.** Relationship between polarization of macrophages and HK3 in GBM. (A) Correlation between HK3 and M1 and M2 macrophages via the TIMER database; (2)Correlation between HK3 and macrophage M2 polarisation-related genes via the TIMER database.

**Figure S4**

**
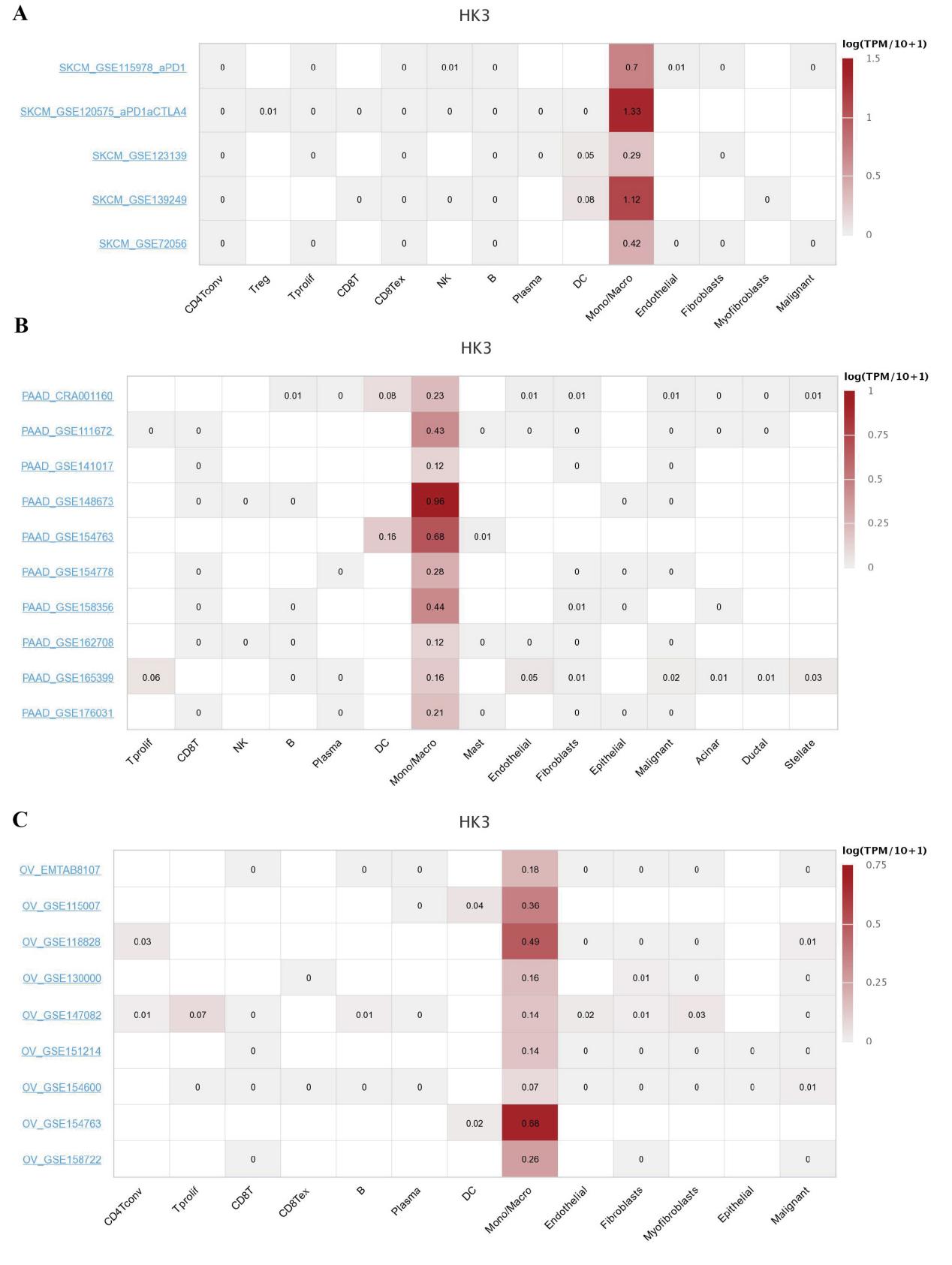
**

**Figure S4.** The HK3 expression in immune cells at the single-cell level through the TISCH database. (A) skin cutaneous melanoma; (B) Pancreatic adenocarcinoma; (C) Ovarian serous cystadenocarcinoma.

**Figure S5**

**
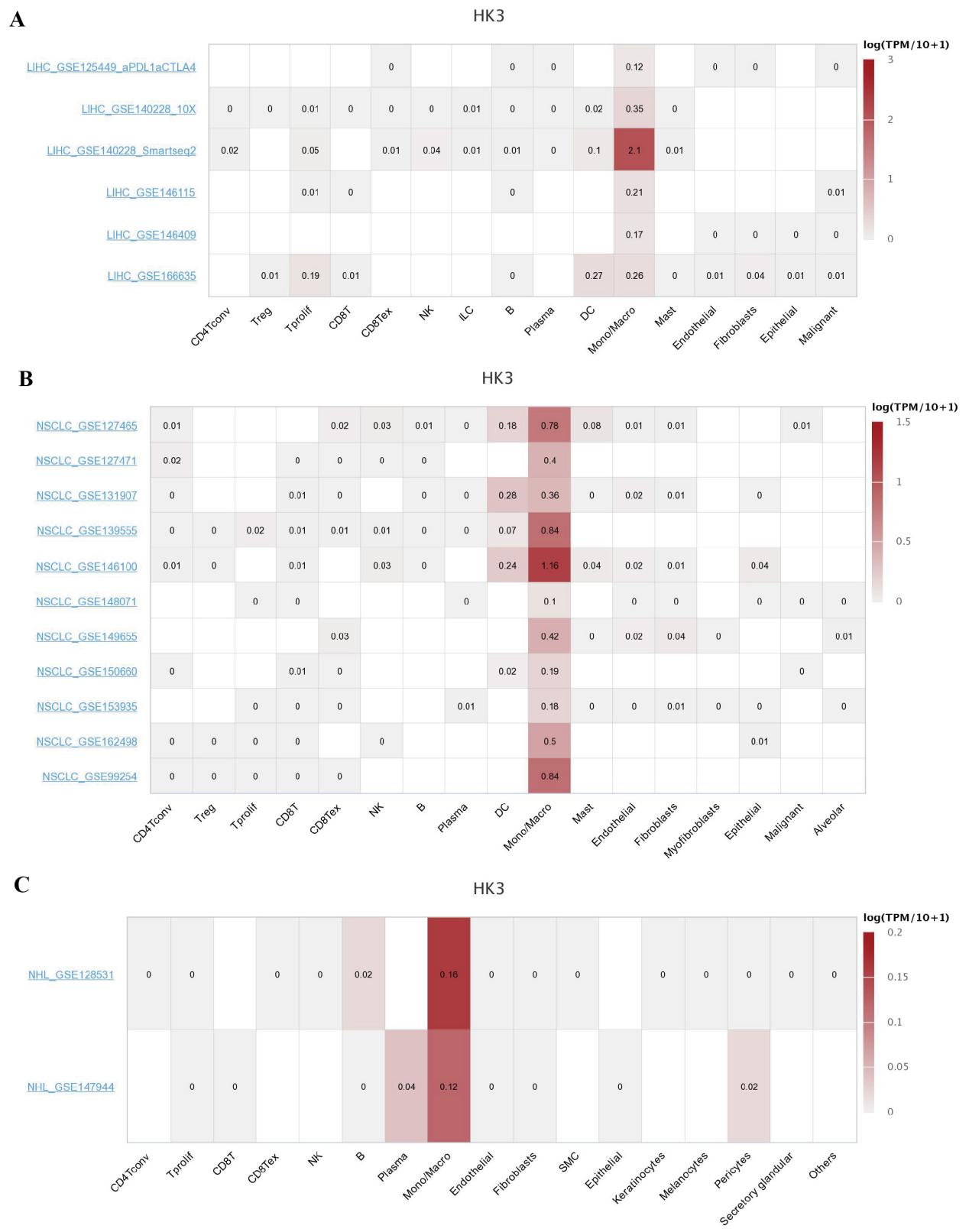
**

**Figure S5.** The HK3 expression in immune cells at the single-cell level through the TISCH database. (A) Liver hepatocellular carcinoma; (B) Non-small cell lung cancer; (C) Non-Hodgkin lymphoma.

**Figure S6**

**
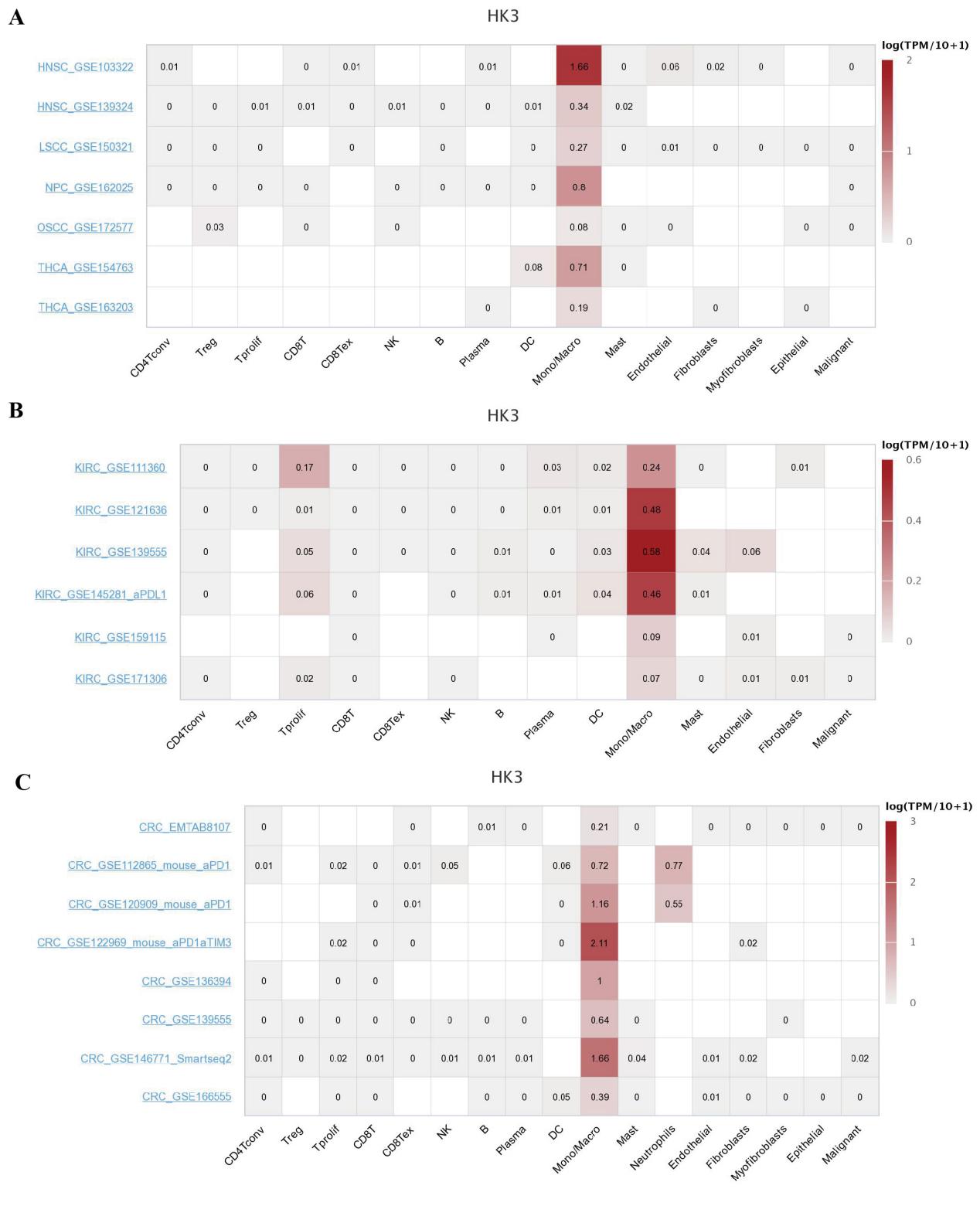
**

**Figure S6.** The HK3 expression in immune cells at the single-cell level through the TISCH database. (A) Head and Neck squamous cell carcinoma; (B) kidney renal clear cell carcinoma; (C) carcinoma of colon and rectum.

**Figure S7**

**
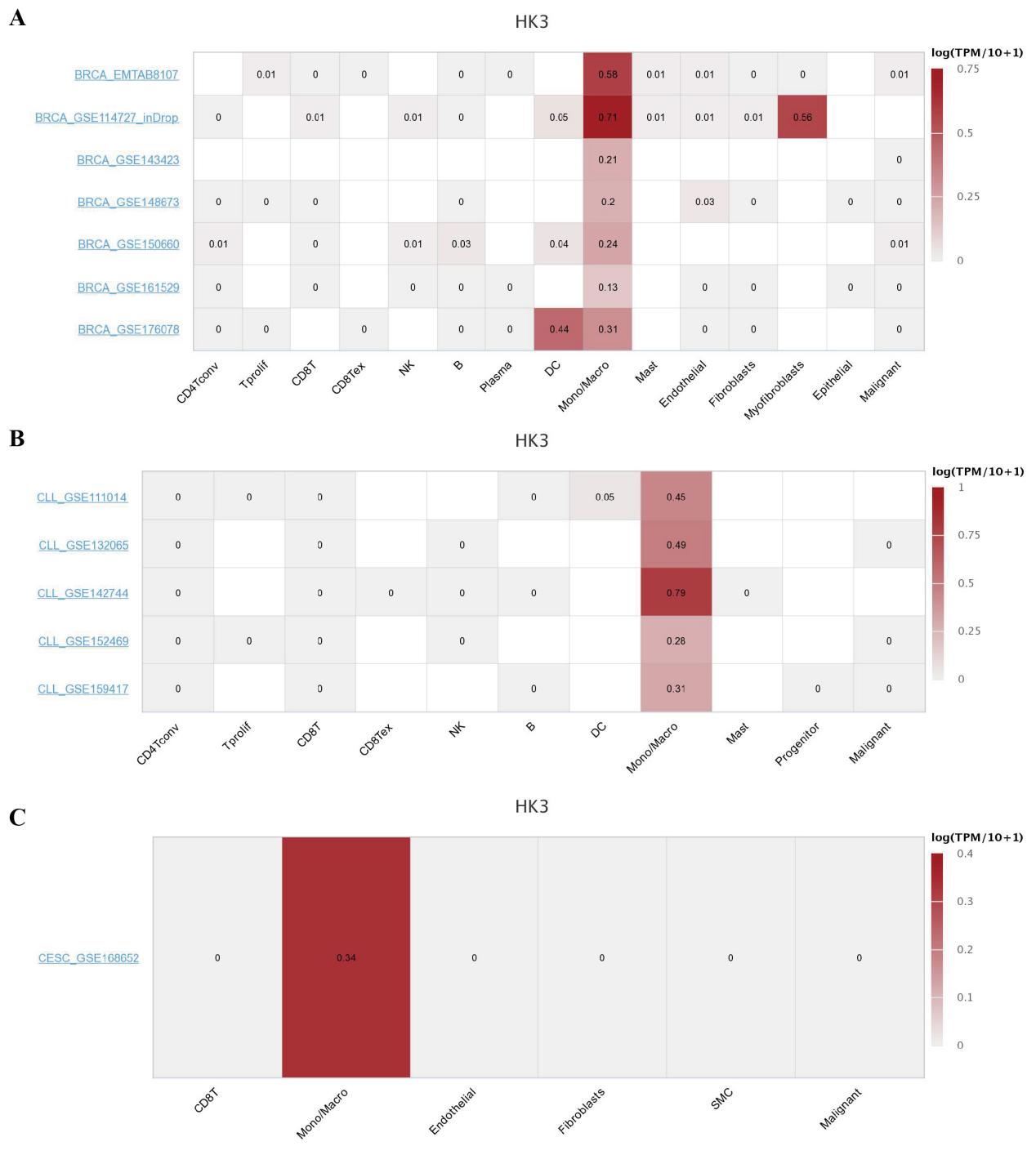
**

**Figure S7.** The HK3 expression in immune cells at the single-cell level through the TISCH database. (A) Breast invasive carcinoma; (B) Chronic lymphocytic leukemia; (C) Cervical squamous cell carcinoma and endocervical adenocarcinoma.
